# Evaluation of Relationship between Sever Early Childhood Caries and Breast Milk`s Lactose among 12- to 24-month-old Children

**DOI:** 10.30476/DENTJODS.2021.91254.1567

**Published:** 2022-09

**Authors:** Hamidreza Poureslami, Maryam Sharifi, Mahla Vahedi, Salehe Sabouri, Parnian Poureslami, Naghmeh Satarzadeh, Nima Hatami, Parisa Jafari

**Affiliations:** 1 Dept. of Pediatric Dentistry, Kerman Dental Faculty, Kerman, Iran; 2 Kerman Health Center, Kerman University of Medical Sciences, Kerman, Iran; 3 Neuropharmacology Institute, Kerman University of Medical Sciences, Kerman, Iran; 4 Postgraduate Student, Dental Faculty, Kerman University of Medical Sciences, Kerman, Iran; 5 Faculty of Pharmacy, Kerman University of Medical Sciences, Kerman, Iran; 6 Dept. of Endodontics, Dental Faculty, Kerman University of Medical Sciences, Kerman, Iran; 7 Postgraduate Student, Dept. of Pediatric Dentistry, School of Dentistry, Kerman University of Medical Sciences, Kerman, Iran

**Keywords:** Breastfeeding, Lactose, Early Childhood Caries, Breast milk

## Abstract

**Statement of the Problem:**

Severe early childhood caries (S-ECC) is common among infants and toddlers. It has many problems and challenges for families as well as dentists in fields of prevention and treatment.

**Purpose:**

The study aimed to investigate the relationship between occurrence of S-ECC and level of lactose in breast milk, in infants aged 12 to 24 months.

**Materials and Method:**

This cross-sectional descriptive-analytical study was carried out from April to July 2020 on 30 children aged 12 to 24 months with or without S-ECC, who were sol-ely breastfed; their mothers answered questions about their breastfeeding habits. After the child's dental visit, the mother was asked to express 10 to 20ml of her milk as a sample and give it to the researchers. The samples were immediately kept at -4°C and then they were tested for lactose measurement. Finally, the test results were analyzed by SPSS 21 software using independent t-test.

**Results:**

The average amount of lactose in the breast milk of cases with S-ECC infant was 5.74g/100 ml and the average amount of lactose in the breast milk of cases without S-ECC infant was 4.64g/100 ml. There was no significant difference in lactose concentration between the two groups (*p* Value=0.64). The average number of breastfeeding times in cases with S-ECC infants was 7.87 per day while in the healthy cases this was 7.33 per day. There was no significant difference between the numbers of breastfeeding times per day in two groups.

**Conclusion:**

According to this study, the amount of lactose in breast milk of children with S-ECC was slightly higher than the amount of lactose in breast milk in children with healthy teeth.

## Introduction

Early childhood caries (ECC) is a non-communicable but transmissible infectious disease. Caries usually progresses to the pulp and causes pain, eventually leading to the formation of dental abscesses that adversely affect the child's health [ 1 ]. ECC means the presence of one or more decayed tooth surfaces in each tooth of a child aged 71 months or less. Severe early childhood caries (S-ECC) is a severe type of ECC that is characterized by the age of less than three years [ 2 ]. Various factors affect the initiation and continuation of the disease, such as food substrate and acid-producing bacteria, which form a biofilm that adheres to the tooth surface [ 3 ]. Over time, the substrate acts as a bacterial nutrient and the acid are produced by bacteria [ 3 - 4 ]. There are many predisposing factors for this disease such as breastfeeding at night, diet with high sugar content, poor parental awareness of the child's oral health, low socioeconomic level of the family, and lack of access to oral care centers [ 4 - 6 ]. If performed in an inappropriate way, breast-feeding can be a predisposing factor for S-ECC [ 5 - 6 ]. Some factors including the lactose level in breast milk or formula, and contiguity of the milk with the erupted teeth, determine the risk of dental decay [ 7 - 9 ]. Breastfeeding is beneficial for both mother and baby. Unlike formula, breast milk contains casein, which prevents the growth and adhesion of cariogenic bacteria, especially streptococci, to the enamel [ 10 ]. Human breast milk has the highest amount of lactose compared to other species (7 grams per 100ml). Lactose is converted to glucose and galactose before intestinal absorption [ 11 ]. Mohebbi *et al.* [ 1 ] evaluated the eating habits of children and its effect on ECC in communities that were normally breastfed for a long time. According to their findings, breast milk was the cause of ECC due to its high lactose content [ 1 ]. Branger *et al.* [ 12 ] reviewed articles that assessed the association between breastfeeding and S-ECC. Prevalence of ECC has been reported up to 71% in the world [ 13 ]. In Iran, the prevalence of S-ECC in different regions has been reported 10% to 45% [ 13 - 15 ]. Therefore, this study was conducted due to the high occurrence of S-ECC in Iran and many countries, and a likely relationship between prevalence and severity of S-ECC with the amount of breast milk lactose.

## Materials and Method

This study was approved by the Ethics Committee of Kerman University of Medical Sciences with the code IR.KMU.REC.1398.549. The cross-sectional descriptive-analytical study was performed on 30 children of 12 to 24 months with or without S-ECC. The study population was selected from mothers who brought their children to public health centers in Kerman for childhood vaccination as well as to the pediatric dentistry clinics. The mothers were interviewed by third author and they were selected from low socio-economic families because ECC is more prevalent in these families. The children were examined for maxillary anterior teeth in a knee-to-knee position. Clinical examination of children's teeth was performed by disposable oral mirror after drying the surface of maxillary incisors. The presence of any signs of caries on the labial surfaces of the four maxillary incisors was assessed for S-ECC diagnosis, and the presence or absence of caries was noted in the relevant checklist. Inclusion criteria for the mothers were defined as age at delivery between 18 to 45 years, and pregnancy period of 37 weeks and more. The exclusion criteria were defined as maternal diabetes, maternal tobacco, and/or alcohol consumption, feeding by formula.

Mothers were asked to answer the checklist questions while reading and signing the informed consent form for attending in the study. Finally, they extracted 10 to 20 ml of milk by hand or with a special pump to a collection tube. To ensure blindness, all collection tubes were coded as recorded in the consent form and checklist. The samples were immediately placed at -4°C and kept at this temperature until the experiment was performed. On the day of the experiment, the samples were transferred to the laboratory. 

For the measurement of lactose, phenol-sulfuric acid method, which is one of the most common methods for measuring carbohydrates in solution, was adopted. In continue, the spectrophotometer was zeroed with the blank solution and then the wavelength of the samples was read at 490nm. The wavelengths obtained from each sample were recorded. Finally, the lactose concentration was calculated in mg/L. To report values in percentage, (g of lactose in 100 ml of milk) the numbers were divided by 10,000 and the concentrations obtained from the sample were recorded. 

The obtained data were statistically analyzed by a statistical expert using SPSS 21 software. Independent t-test was applied to evaluate the relationship between the lactose level and suffering from ECC.

## Results

In this study, the amount of lactose in 30 human breast milk samples was measured. The children of 15 mothers did not have S-ECC. Their characteristics are given in Table 1, and characteristics of 15 children with S-ECC are given in Table 2. Table 3 shows the comparison of the two groups.

**Table 1:** Distribution of frequency of variables in the group without Sever Early Childhood Caries

Sample Code	Child age in month	Child gender	Amount of lactose in breast milk	Number of feedings in 24 hours
1	16	Girl	5.66	7
2	14	Boy	4.30	6
4	12	Boy	7.01	8
5	19	Girl	1.54	10
6	14	Girl	5.70	7
8	18	Boy	4.50	7
9	16	Girl	1.81	4
10	14	Girl	3.10	8
11	16	Boy	7.07	10
12	23	Girl	4.77	8
14	15	Boy	4.40	2
16	18	Girl	4.57	10
17	13	Boy	3.58	9
18	12	Girl	5.34	6
23	14	Boy	6.27	8

**Table 2:** Distribution of frequency of variables in the group with Sever Early Childhood Caries

Sample Code	Child age in month	Child gender	Amount of lactose in breast milk	Number of feedings in 24 hours
3	23	girl	5.35	10
7	16	boy	7.86	9
13	13	girl	10.24	13
15	13	girl	4.84	12
19	19	boy	8.80	6
20	13	boy	4.87	6
21	14	boy	4.72	15
22	22	girl	6.60	2
24	16	boy	7.20	7
25	23	boy	7.46	5
26	22	boy	5.75	3
27	14	boy	4.34	9
28	16	girl	3.07	7
29	15	boy	2.92	6
30	18	boy	2.10	8

**Table 3:** Amount of Lactose in the breast milk samples in the two groups

Suffering from S-ECC *	Number of children	Average lactose content (SD**)	*p* Value
Yes	15	5.74 (2.28)	0.14
No	15	4.64 (1.65)	
Total	30	5.19 (1.96)	

* S-ECC= Sever Early Childhood Caries

** SD= Standard Deviation

## Discussion

Thirty infants aged 12 to 24 months (mean age 16.4) of both genders who were exclusively breastfed were included in the study with their mothers. Fifteen children had S-ECC. The maximum amount of lactose was 10.24 g per 100ml of milk (belong to the S-ECC group) and the minimum amount was 1.54g/100ml (belong to the non-S-ECC group) (Tables 1 and Table 2). The mean of lactose among 30 mothers was 5.19g/ 100ml (Table 3). A study by Khan *et al.* [ 16 ] reported that the average lactose level in the milk samples of 15 mothers was 6.8g/100ml and the study by Aumeistere *et al.* [ 6 ] has reported this level to be 6.53g/100ml. In both studies [ 6 - 16 ], the amount of lactose in breast milk was higher than the amount of lactose in samples of the present study. These differences can be due to different quality and quantity of nutritional habits in the mothers in different countries. There was no study on the level of lactose in mother breast milk in relation to the occurrence of S-ECC in their children's teeth. In the present study, the amount of lactose in S-ECC group was slightly more than the group of without S-ECC. Khan *et al.* [ 16 ] found that more breast sucking and breastfeeding could lead to an increase the amount of lactose in the milk. In our study, the mean of lactose was slightly higher in the breast milk of mothers who breastfed their baby more frequently than those who breastfed their baby less frequently (Tables 1-Table 2) [ 16 ]. In that study, the average breastfeeding frequency was 11 times in 24 hours whereas in the present study, this average was 7.6 per 24 hours. In both studies, spectrophotometric method was employed to determine the concentration of lactose [ 16 ]. In the present study, we did not assess the amount of fat and protein in the milk samples, but in the Khan *et al.*’s study [ 16 ], it was reported that lack of enough protein in breast milk prevents the baby from getting full. Consequently, this increase in the number of breastfeeding times can eventually cause more caries [ 16 ]. Table 4 shows studies related to breast-feeding and dental caries. 

**Table 4:** The studies related to breast feeding and dental caries

Sample size	Year	Researchers
28	2017	Aumeister *et al.*[ 6 ].
15	2013	Khan *et al.*[ 16 ].
504	2008	Mohebbi *et al.*[ 1 ].
345	2018	Feldens *et al.*[ 8 ].
509	2014	Hong *et al.*[ 9 ].
160	2014	Soto *et al.*[ 10 ].
565	2010	Sugito *et al.*[ 11 ].
35	2019	Branger *et al.*[ 12 ].

Small sample size was a limitation for the present study. This limitation had two reasons including fewer mothers visited clinics due to the spread of COVID-19 and some mothers were reluctant to give their milk samples. Future studies can focus on simultaneous evaluation of the fat and protein levels with lactose level. In addition, mothers’ nutrition can be considered as a factor in the relationship between breast milk’s lactose and S-ECC.

## Conclusion

According to the results of this study, the mean level of lactose in the breast milk samples was lower than the mean level of lactose in breast milk in other countries, and the lactose level in breast milk in children with S-ECC was slightly higher than the lactose level in breast milk in children with healthy teeth.

## Acknowledgement

This article is based on under-graduation thesis, performed at Kerman University of Medical Science, Dental Faculty.

## Conflict of Interests

There is no conflict of interests in this study.
